# Mutual inhibition between HDAC9 and miR-17 regulates osteogenesis of human periodontal ligament stem cells in inflammatory conditions

**DOI:** 10.1038/s41419-018-0480-6

**Published:** 2018-04-24

**Authors:** Liya Li, Wenjia Liu, Hong Wang, Qianjuan Yang, Liqiang Zhang, Fang Jin, Yan Jin

**Affiliations:** 1 State Key Laboratory of Military Stomatology & National Clinical Research Center for Oral Disease, Center for Tissue Engineering, School of Stomatology, The Fourth Military Medical University, 710032 Xi’an, Shaanxi China; 2 Xi’an Institute of Tissue Engineering and Regenerative Medicine, 710032 Xi’an, Shaanxi China; 3 State Key Laboratory of Military Stomatology, Department of Orthodontics, School of Stomatology, The Fourth Military Medical University, 710032 Xi’an, Shaanxi China

## Abstract

Histone deacetylases (HDAC) plays important roles in the post-translational modifications of histone cores as well as non-histone targets. Many of them are involved in key inflammatory processes. Despite their importance, whether and how HDAC9 is regulated under inflammatory conditions remains unclear. The aim of this study was to evaluate the effects of HDAC9 under chronic inflammation condition in human periodontal ligament stromal cell (PDLSCs) and to explore the underlying regulatory mechanism. PDLSCs from healthy or periodontitis human tissue was compared. The therapeutic effects of HDAC inhibitors was determined in PDLSC pellet transplanted nude mice and LPS-induced rat periodontitis. We report that HDAC9 was the most affected HDAC family member under inflammatory conditions in PDLSCs. HDAC9 impaired osteogenic differentiation capacity of PDLSCs under inflammatory conditions. Downregulation of HDAC9 by HDAC inhibitors or si-HDAC9 rescued the osteogenic differentiation capacity of inflammatory PDLSC to a similar level with the healthy PDLSC. In this context, HDAC9 and miR-17 formed an inhibitory loop. The inhibition of miR-17 aggravated loss of calcified nodules in inflamed PDLSCs and interrupted the effect of HDAC inhibitor in rescuing osteogenesis. In vivo experiments using nude mice and LPS-induced periodontitis model confirmed that HDAC inhibitors could improve new bone formation. We conclude that HDAC inhibitors improved osteogenesis of PDLSCs in vitro and periodontitis in vivo.

## Introduction

During the development of periodontitis, a complicated bacteria community forms biofilms and leads to the succeeding cytotoxic infiltrating immune response. The persistent immune response leads to tissue damage and bone loss due to increased numbers of osteoclast and decreased numbers of osteoblast^1,2^. Specifically, the memory of periodontitis is transferable to generations of cells, which is a classic characteristic of the involvement of epigenetics^3^. Thus, we used periodontitis as a model to study the inflammatory regulated epigenetic mediators.

HDAC controls gene expression by enzymatic removal of the negatively charged acetyl groups from the positively charged lysine on histone tails, resulting in tightening the negatively charged DNA backbone and preventing its access for transcription. Previous studies reported that HDACs can be activated in response to persistent inflammatory signals during pneumonia, rheumatoid arthritis, hemorrhagic cystitis, and other diseases, driving the balance toward inflammation rather than resolution^4–6^.

Several studies of a direct relationships between HDAC9 and inflammatory diseases had been found in recent 10 years. For example, Yan K. found that HDAC9 deficiency in a CD4+ T cell-mediated autoimmunity mice had decreased inflammation and produced less cytokine and chemokine due to increase of PPAR-γ^7^. In addition, HDAC9 is highly expressed in Treg cells. Knockout of HDAC9 alone increased resistance to dextran sulfate sodium-induced colitis and increased the number of Treg cells^8^. Furthermore, HDAC9-deficient Treg cells proliferate faster and leads to stronger immune repression^9^. Taken together, HDAC9 act as a strong immune enhancer.

Regarding to potential epigenetic mediators in periodontitis, the RNA expression level of HDAC 1, 5, 8, 9 was upregulated in gingival tissue of most periodontitis patients compared with mild inflamed patients^10^. Particularly, HDAC9 was exclusively expressed in the perivascular regions in a discontinuous pattern^10^. According to our knowledge, perivascular cells which plays key role to periodontal tissue differentiation is likely to be mesenchymal stem cells. Thus, we hypothesized that HDAC9 caused aberrant differentiation of mesenchymal stem cells may be key to solving inflammatory impaired bone remodeling diseases.

HDI act by competitively binding to zinc finger region of HDAC protein and widely blocking HDAC containing multi-protein machinery^11,12^. Despite its wide blockade of HDAC family members, some report that roles of HDI include specifically participating in promoting osteogenic differentiation. For example, HDI promotes terminal osteoblast differentiation and extracellular matrix production and bone regeneration^13,14^, and modulate inflammatory responses^15^ in bone remodeling-related diseases such as rheumatoid arthritis, myeloma bone disease^16^. However, the potential of these HDI to treat periodontitis has not been proved yet and the regulatory mechanisms of HDACs remains unclear.

The tooth developmental network is regulated by many miRNAs and they participate in the differentiation, repair, and regeneration of dental cells by differential expression in dental tissues^17^. In addition, treatment of inflammatory cytokines to periodontal ligament cells results in expressional changes of various miRNAs, such as miR-138, miR182^18,19^, suggesting that miRNAs which regulate periodontal tissue development and repair may be affected by inflammatory environmental cytokines and could result in impaired periodontal tissue regeneration. miR17-92a cluster is first described in 2001^20^ in mammalians, known as tissue-specific expressed onco-miR, forms signaling loop with myc protein, miR17-92a regulates more than a hundred targets involved in proliferation depending on different cellular context, their role in affecting the HDAC, which is responsible for the global proliferation inhibition remains unknown^21^. miR-17 in periodontal ligament stem cells targets the 3′ untranslated regions of a Smad ubiquitin regulatory factor one(Smurf1), which when activated under chronic inflammation, would lead to increased degradation of various osteoblast-specific factors^3^. These evidences prompt us to verify that if miR-17-92a could regulate HDAC modulated dental tissue differentiation under inflammatory conditions.

In the article, we showed that HDAC9 is the most affected HDAC family member in inflammatory PDLSCs. We discovered that the onco-miR, miR-17 is a member of the epi-miRNAs. miR-17 induced osteogenesis of inflamed periodontal adult stem cell through inhibition of HDAC9. HDAC inhibitor NaB improved differentiation of PDLSCs in vitro and periodontitis in vivo. mir-17 and HDAC9 negatively affect each other under chronic inflammatory conditions in the adult stem cell in tooth tissue. Finally, we revealed that the rescue of osteogenesis by HDAC inhibitor depended on miR-17.

## Results

### Inflammation impaired the osteogenesis capacity of PDLSCs

Since adult stems cells have long been recognized as a critical population in restoring tissue function under inflammatory conditions, we asked if inflammation affects PDLSCs, the adult stem cells from periodontal ligament tissue and what specific effects has inflammation brought to PDLSCs.

**Fig. 1:**
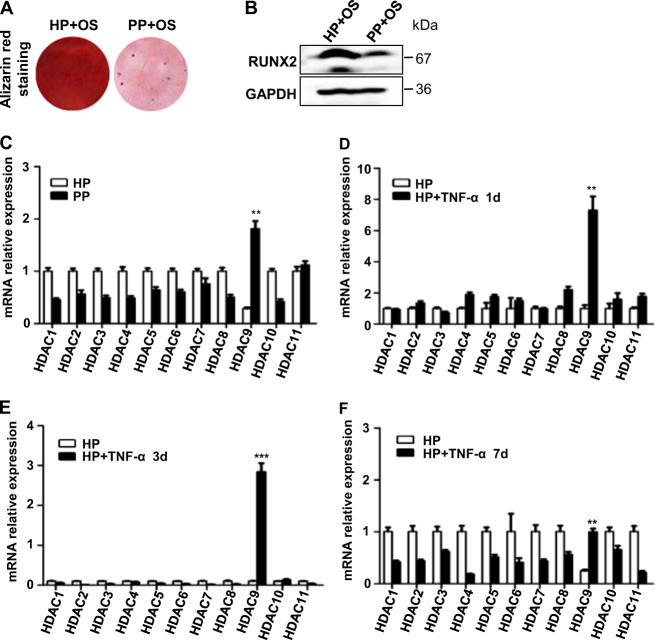
Inflammation impaired the osteogenesis capacity of PDLSCs and led to expressional changes of HDAC9 in PDLSCs. **a** H-PDLSCs (HP) and P-PDLSCs (PP) stimulated for 4 weeks with osteogenic induction (OS) was stained by Alizarin red. **b** Protein expression level of osteoblast marker Runt-related transcription factor 2 (RUNX2) in H-PDLSCs and P-PDLSCs stimulated for 7 days with osteogenic induction was measured by Western blot. **c** RNA expression level of HDAC family members in cultured H-PDLSCs and P-PDLSCs was measured by RT-qPCR. **d**–**f** RNA expression level of HDAC family members in cultured H-PDLSCs and P-PDLSCs was measured by RT-qPCR after TNF-α stimulation for 1, 3, or 7 days. *p* < 0.05 was considered statistically significant (NS, *p* > 0.05, *p < 0.05, **p < 0.01, ***p < 0.001)

PDLSCs isolated from periodontal tissue of healthy individual (H-PDLSCs) and PDLSCs isolated from periodontitis patients (P-PDLSCs) were compared. The cells were examined by definitions of MSC from the International Society for Cell Therapy. They were plastic-adherent and have fibroblast like morphology when cultured (Supplementary Fig. 4). They can be differentiated to osteoblast, and adipocytes when stimulated (Fig. 1a–d). Flow cytometry revealed that H-PDLSCs were positive for Mesenchymal stromal cell surface markers, CD105 (87.2%), CD90 (93.6%), and CD146 (25.5%), and negative for hematopoietic progenitor cell surface marker CD34 (2.4%) and leukocyte common antigen CD45 (2.6%) (Supplementary Fig. 1A). Similarly, P-PDLSCs were positive for CD105 (85.90%), CD90 (99.74%), CD146 (78.39%), and negative for CD34 (1.69%), CD45 (0.78%) (Supplementary Fig. 1B).

To explore the effects of chronic inflammation to PDLSCs in periodontitis, their proliferation rate and osteogenic differentiation potential were examined. During 7 days of MTT measurement, the proliferation rate of P-PDLSCs and H-PDLSCs were not affected by addition of HDI (Supplementary Fig. S3). Interestingly, a significant higher level of the proliferation rate of P-PDLSCs appeared at day 3, compared to H-PDLSCs (Supplementary Fig. 2A–F). After osteogenic stimulation, P-PDLSCs showed decreased protein expression level of the master osteoblastic differentiation transcription factor RUNX2 compared to H-PDLSCs (Fig. 1b). The Alizarin red staining were consistent with the results of RUNX2 (Fig. 1a). Taken together, we showed that PDLSCs from periodontitis patients bared decreased osteogenic differentiation potentials and presented an unhealthy rapid proliferation instead.

### Inflammation leads to epigenetic changes in hPDLSCs and HDAC9 is involved

The presence of epigenetic regulation in periodontitis is confirmed by measuring inflammation in different passages of PDLSCs derived from periodontitis patients. Previous results of our laboratory have shown that even without stimulation of inflammatory cytokines, the cultured P-PDLSCs presented persistent differentiation pattern compared with that of the H-PDLSCs^16^.

To verify if histone modifications is involved in impairing periodontal tissue differentiation under inflammatory conditions, we first compared expression of histone modification effectors HDACs in H-PDLSCs, P-PDLSCs, and TNF-α-stimulated H-PDLSCs. The screening of HDAC family showed that the RNA expression level of HDAC9 had been markedly increased in P-PDLSCs and TNF-α-stimulated H-PDLSCs (Fig. 1c–f). This result is further verified by decreased protein expression level of HDAC9 in P-PDLSCs, suggesting that histone acetylation by HDAC9 is associated in PDLSC-derived periodontal tissue regeneration under inflammatory conditions (Supplementary Fig. S6).

### Inhibition of HDAC9 restores osteogenic differentiation of P-PDLSCs

**Fig. 2:**
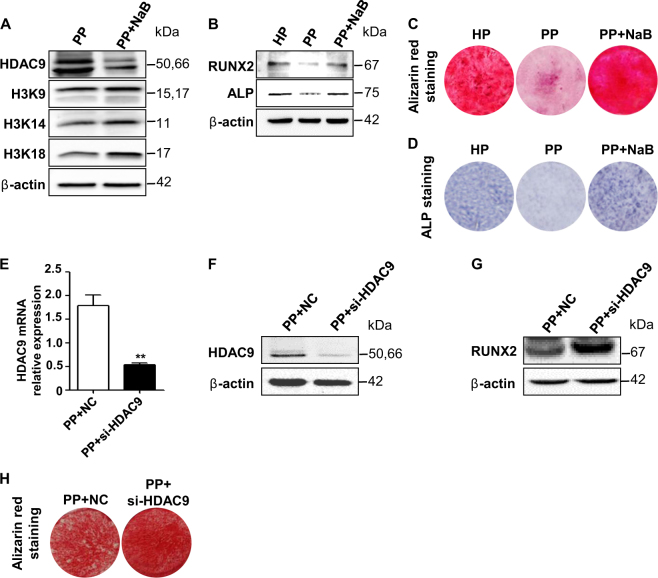
Inhibition of HDAC family by HDI or specific inhibition of HDAC9 by si-HDAC9 rescued osteogenesis of P-PDLSCs. **a** Protein expression level of HDAC9 and well-known histone targets of HDAC9 in untreated, NaB-treated P-PDLSCs. **b** Protein expression level of osteogenic markers RUNX2 and alkaline phosphatase (ALP). Comparison of osteogenesis in H-PDLSCs, P-PDLSCs, or NaB-treated P-PDLSCs 4 weeks after osteogenic induction by Alizarin red staining (**c**) or 7 days after induction by ALP staining (**d**). **e**–**f** RNA and protein expression levels of HDAC9 was analyzed between P-PDSLCs and si-HDAC9-treated P-PDLSCs. Analysis of protein expression level of osteogenesis marker RUNX2 (**g**) and calcified nodules by Alizarin red staining (**h**)

To verify if inhibiting HDAC9 expression could rescue inflammatory impaired osteogenic differentiation of PDLSCs, HDAC9 is interfered by NaB and si-HDAC9. Treatment of NaB downregulated protein expression level of HDAC9, and increased the acetylation of several well-known HDAC9 histone targets (Fig. 2a). Downregulation of HDACs with appropriate concentration of NaB restored the osteogenic differentiation capacity and expression of osteogenic markers, suggesting a critical role of HDACs in impairing P-PDLSC osteogenesis (Supplementary Fig. S7, Fig. 2b–d). In addition, specifically downregulated HDAC9 by si-HDAC9 improved osteogenic differentiation of P-PDLSCs, as revealed by protein expression of osteogenic markers RUNX2 and Alizarin red staining (Fig. 2e–h).

### mir-17 and HDAC9 negatively inhibit each other in regulation of osteogenic differentiation of PDLSCs in vitro

**Fig. 3:**
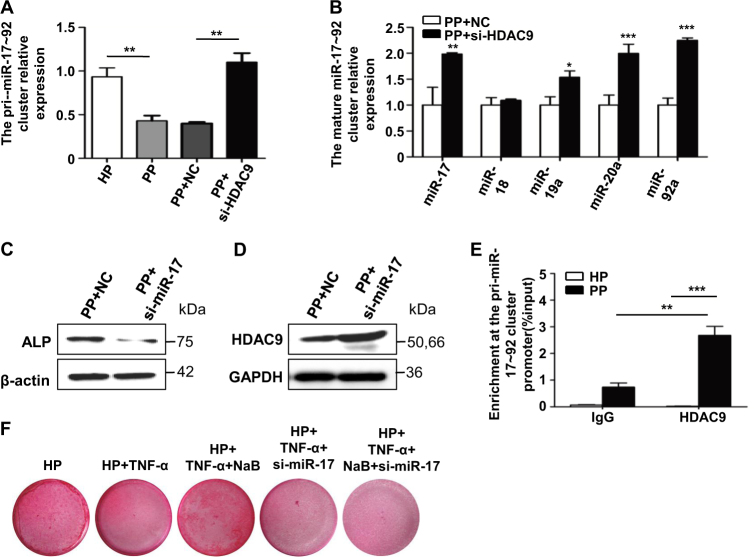
miR-17 and HDAC9 negatively inhibit each other in regulation of osteogenic differentiation of PDLSCs in vitro. **a** Comparison of the RNA expression level of pri-miR17-92a cluster between H-PDLSCs, P-PDLSCs, and si-HDAC9-treated P-PDLSCs. **b** Comparison of the RNA expression of mature miR-17-92a between P-PDLSCs and si-HDAC9-treated P-PDLSCs. Comparison of the protein expression level of ALP (**c**) and HDAC9 (**d**) between P-PDLSCs and miR-17 inhibitor-treated P-PDLSCs. **e** Analysis of the enrichment of HDAC9 protein at the pri-miR-17-92a cluster promoter region by Chromatin Immunoprecipitation (ChIP). **f** Analysis of calcified nodules by Alizarin red staining in NaB, si-miR-17, or NaB plus si-miR-17-treated TNF-α-stimulated PDLSCs

To answer how HDAC is regulated in PDLSCs under inflammatory conditions, epi-miRNAs, which is known to regulate the expression of HDAC or HDAC drove histone modifications with an association to teeth development, was reviewed. miR-17-92 family is important in proliferation of PDLSCs under chronic inflammatory conditions. We first examined the association of HDAC9 with miR-17~92a in PDLSCs of periodontitis patients. The expression of pri-miR-17~92a was downregulated in P-PDLSCs compared to H-PDLSCs (Fig. 3a). Interestingly, downregulation of HDAC9 by si-HDAC9 in P-PDLSCs restored the expression of pri-miR-17-92a as well as the mature miR17-92a, though, miR-18 was not affected, suggesting that HDAC9 inhibited miR17-92a (Fig. 3a, b). This is surprising since HDAC9 promotes proliferation and miR-17~92a belongs to the onco-miR family. Furthermore, inhibition of miR-17 by anti-miR-17 oligonucleotides (si-miR-17) resulted in the upregulation of the protein expression level of HDAC9 (Fig. 3d). Taken together, the miR-17 and HDAC9 formed inhibitory loop under inflammatory conditions. ChIP experiments revealed the promoter region of miR-17-92a HDAC9 enrichment in P-PDLSC samples, suggesting that HDAC9 inhibits the expression of miR17-92a by direct deacetylation (Fig. 3e).

### miR-17 is essential for NaB to rescue the osteo-differentiation of TNF-α-stimulated PDLSCs

In order to find out if miR-17 plays important role in the inhibitory loop in osteogenesis of PDLSCs, we examined the effects of miR-17 on osteogenesis of PDLSCs. When inhibit miR-17 in TNF-α-stimulated PDLSCs, protein expression level of osteogenic marker alkaline phosphatase (ALP) was downregulated (Fig. 3c). According to previous results in our lab, overexpression of miR-17 decreased expression levels of osteogenic markers and bone matrix formation, inhibition of miR-17 alone increased osteoblast marker genes, suggesting that miR-17 is a negative regulator of osteogenic differentiation in H-PDLSCs^3^. Furthermore, miR-17 became a positive regulator of osteogenic differentiation in P-PDLSCs^3^. Consistent with these results, in the current study, Alizarin red staining showed that downregulation of miR-17 inhibited calcified nodule formation in TNF-α-stimulated H-PDLSCs (Fig. 3f). Furthermore, simultaneous addition of si-miR-17 and NaB inhibited osteogenesis to a similar extent than using si-miR17 alone in TNF-α-stimulated H-PDLSCs, suggesting that the rescue of osteogenesis by NaB largely depended on the expression of miR-17 (Fig. 3f).

### NaB rescues osteo-differentiation in vivo

**Fig. 4:**
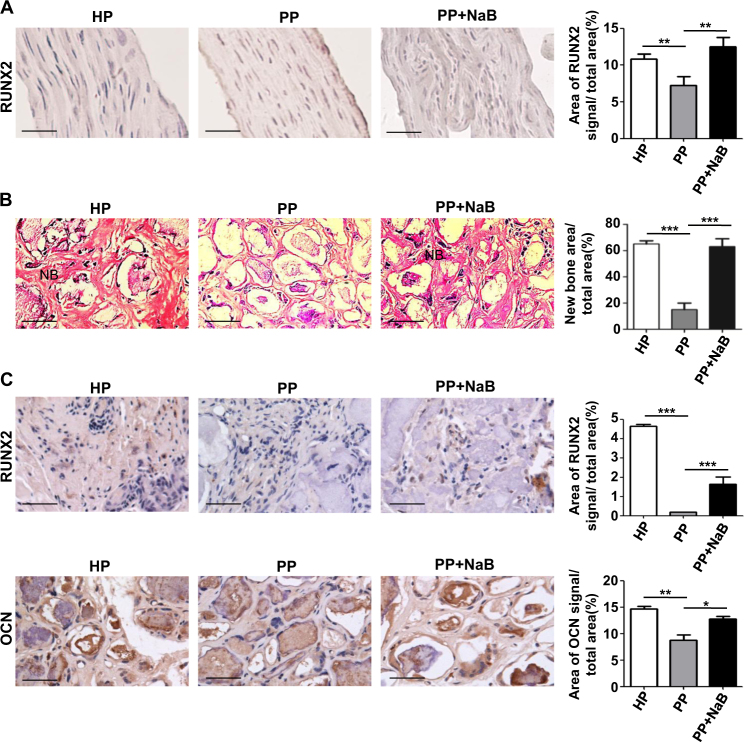
NaB ameliorated LPS-induced crestal bone loss in SD rat in vivo. **a** Schematic illustration of the LPS-induced periodontitis model in SD rats. Twelve 8-week-old SD rats were randomly distributed into four groups with three rats per group. 10 μl drugs were injected in each group into the maxillary palatal gingiva between the first and second upper molars and were repeated every other day on 3 separate days. All rats were anesthetized and euthanized by exsanguination on day 7. **b** Representative micro CT reconstruction images of the alveolar bone loss of SD rat, LPS-treated SD rat, or LPS plus NaB-treated SD rat and quantitative analysis of the crestal bone loss (*n* = 3). **c** Representative micro CT reconstruction images of the bone changes at the drug injection site

To explore whether NaB is able to rescue inflammation impaired osteogenesis in vivo, LPS-induced periodonitis SD rat model was treated with normal saline or NaB (*n* = 3). The LPS and normal saline-treated LPS group exhibited obvious alveolar bone loss and a high bone resorption (Fig. 4a–c). In contrast, the NaB-treated group showed less alveolar bone loss and minor resorption (Fig. 4a–c). Thus, NaB significantly improved periodontitis-derived alveolar bone loss.

**Fig. 5:**
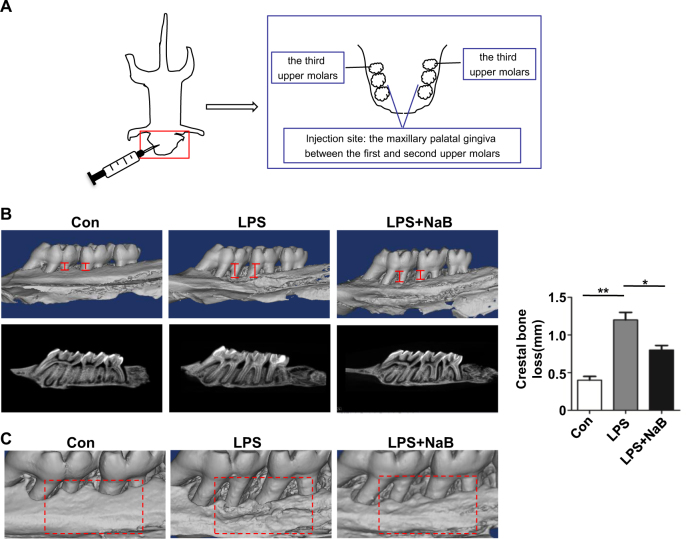
Identification of the osteogenic capacity of NaB-treated P-PDLSCs pellets in vitro and of the osteogenic differentiation in the cell pellet that transplanted in nude mice. **a** IHC-P staining of RUNX2 in H-PDLSCs, P-PDLSCs, or NaB-treated P-PDLSCs cell pellet before transplanted to immunocompromised mice (left panel). Scale bars represent 50 μm. Statistical quantification of RUNX2 signal by ImageJ IHC image analysis toolbox plugin (right panel). **b** Influence of NaB on bone formation was evaluated by hematoxylin and eosin (H&E) staining after subcutaneous transplantation into immunocompromised mice (left panel). Scale bars represent 50 μm. Quantitative analysis results of the H&E staining of the transplants (right panel). **c** IHC-P staining of RUNX2 and OCN in H-PDLSCs, P-PDLSCs, or NaB-treated P-PDLSCs cell pellets after transplanted to immunocompromised mice (left panel). Scale bars represent 50 μm. Statistical quantification of RUNX2 and OCN signal (right panel)

**Fig. 6:**
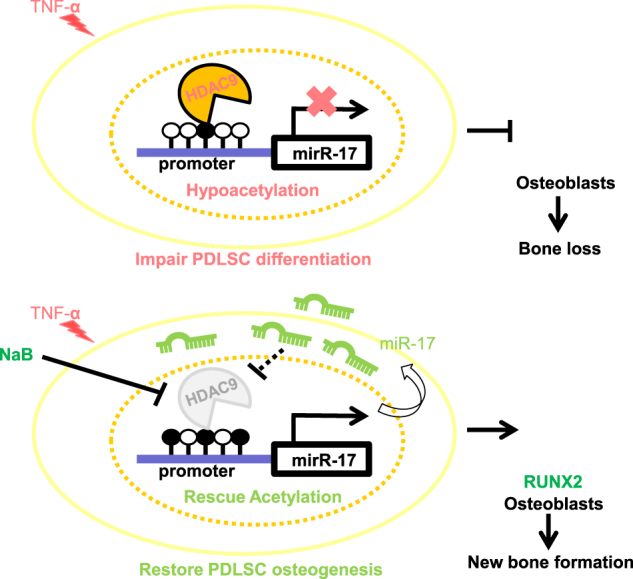
Schematic illustration of the relationship between HDAC9, miR-17, and bone regeneration. The mutual inhibition between HDAC9 and miR-17 in P-PDLSCs regulates the osteogenesis of PDLSCs and affects the bone regeneration in inflammatory condition

To further explore whether inhibiting HDACs in PDLSCs alone would improve the inflammation impaired bone formation, cell pellets of PDLSCs enwrapped with hydroxyapatite were dorsal subcutaneous transplanted in immunocompromised mice. Among them, cell pellets of H-PDLSCs were taken as positive control and cell pellets of P-PDSLCs treated with or without NaB were compared (Fig. 5a–c). Before implantation, cell pellets of NaB-treated P-PDLSCs exhibited higher expressional level of RUNX2 (Fig. 5a). After implanted for 56 days, the NaB-treated P-PDSLCs pellet showed comparable area of new bone-like tissue formation with the H-PDLSC pellet (Fig. 5b). In contrast, the P-PDLSC pellet had little or no new bone-like tissue formed (Fig. 5b). Specifically, the expressional level of osteogenic marker RUNX2 was elevated in the nuclear and the binding of secreted OCN to hydroxyapatite also increased (Fig. 5c). These results together suggest that inhibition of HDACs in PDLSCs is sufficient to promote periodontal osteogenesis (Fig. 6).

## Discussion

One of the key characteristics of chronic inflammation is its persistence. Removal of inflammation stimuli alone cannot block the progression of bone loss, because inflammation has epigenetically impaired the differentiation of the adult stem cells. Instead of direct targeting inflammation itself, we focused on repairing inflammation impaired adult stem cell in this study. Chronic inflammation impaired the differentiation of MSC, the very important type of adult stem cell for tissue repair. We found that MSC is epigenetically modified under chronic inflammation and the elevated acetylation plays key role in impairing MSC differentiation and proliferation.

Histone modifications is often associated with long-term events. This characteristic make histone modification a pivotal event in various physiological and pathological processes, for example, cell fate determination and diseases such as inflammation and cancer. However, how the not easily reversible histone modification is regulated in response to environmental signals remains largely unknown. Previous reports suggest that epigenetic changes of cells respond to chronic inflammation, as is the case in periodontitis^16^. Periodontal cells are immersed in inflammatory periodontal cytokines secreted by surrounding cells, and will ultimately lead to degeneration of periodontal stem cells which play key role in periodontal tissue regeneration in periodontitis. Due to epigenetic changes, chronic inflammation is not easily reversed. In our study, we showed that histone modifications are involved in periodontitis and we showed mi-RNA participation in the regulation of HDAC9 under inflammatory conditions and rescued the tooth loss in periodontitis by HDI.

HDI were intensely studied as the cognitive enhancers and anti-cancer agents^22,23^. As an anti-cancer agent, the role of HDI in inducing cell cycle arrest and cancer cell death grasped more attention. On the other hand, HDI were reported to have therapeutic roles in the inflammatory diseases through enhancing regulatory T cell function^24–26^. In our study, HDI enhanced the osteogenic differentiation of the adult stem cell under inflammatory conditions. These studies as well as our study put new evidences to a very different role of HDI in cell differentiation.

HDAC4, 5, 7, 9 belong to Class IIa depending on the highly conserved homology. Their post-translational modifications determine the cellular distribution, protein structures, interactions, and localizations. The phosphorylation sites around nuclear localization sequence regulates the shuttle of HDAC protein from cytoplasm to nuclear, and regulates the binding sites responsible for HDAC protein interactions. Interestingly, the enzymes and phosphorylation sites share similarities within HDAC5, 7, and 9, indicating that the Class IIa HDACs is responsive to similar environmental stimulations and may participate in similar biological process. During skeletal development, HDAC7 is responsive to bone morphogenic proteins (BMP2) and can release the transcription of RUNX2. HDAC4 regulates chondrocyte hypertrophy through inhibition of RUNX2^27^. Notably, the Class IIa HDACs vary in cellular distribution. Previously, HDAC9 is found highly expressed in cancer cells^28,29^, immune cells^30^, osteoblast^31^, and a MSC cell line 3A6^31^. The HDAC9 binding protein myocyte enhancer factor-2 interacting transcriptional repressor accelerates osteogenesis and attenuates adipogenesis through quenching PPARγ-2^31^. We further complement the story of HDAC9 and MSC by first identifying HDACs expression in PDLSC and finding that HDAC9 participates in osteogenic differentiation through interacting with miR-17 to repress RUNX2 transcription.

Evidences of miRNAs that regulate key epigenetic effectors involved in histone modifications, such as DNMTs, HDACs, or polycomb genes, have been accumulating in the last few years. Those miRNAs, we call them the epi-miRNAs, includes, for example, miR-148a, miR152, miR222 that targets mRNA of DNMTs and leads to re-expression of hyper-methylated tumor suppressors^32^. The miRNAs clusters, miR-17-92a, miR-106b-25, and miR-106a-363, have been found to control EZH2 expression, which is involved in H3K27me3-mediated tumor suppressor genes in cancer^32^. In our study, we showed that miR-17 is a new member of the epi-miRNA which inhibited the protein expression level of HDAC9. Furthermore, the transcription of miR-17 itself is regulated via deacetylation by HDAC9 in the promoter regions under inflammatory conditions.

Interestingly, the role of miR-17 in PDLSC differentiation can be shifted to either promotion of osteogenesis or inhibition of osteogenesis^16^. In previous studies, miR-17 promotes proliferation in the regulation of B cell lymphoma growth and retinoblastoma, and thus might delay differentiation^15,26^. According to our results, the overall role of miR-17 is likely to be closely associated with the expressional level of HDAC9. When HDAC9 is inhibited by HDI, miR-17 has an inhibitory role in osteogenesis of PDLSC (data not shown). This may explain why a co-inhibit relationship is needed between HDAC9 and miR-17. In the physiological conditions, miR-17 as well as HDAC9 forms an inhibitory balance to regulate the differentiation of PDLSCs and affect adjacent cells to regulate bone formation. However, the mechanisms underlying the shift of the role of miR-17 in osteogenesis needed further exploration.

We demonstrated that the inhibition of HDAC by NaB downregulated miR17-92a family and partially rescued inflammation impaired osteogenesis in vitro and in vivo. We found a new epi-miRNA, the miR-17, which forms a reciprocal signaling loop with HDAC9 in PDLSCs under inflammation condition. Their mutual interaction presents a novel way to interfere with the epi-genome, which can be inherited to generations of cells, by targeting some of the epi-miRNAs. Inhibition of HDAC by NaB as well as si-miR-17 rescues osteogenesis of the human inflammatory PDLSCs. NaB may serve as new therapeutic targets for periodontitis, inflammatory diseases and we provide novel evidences for the involvement of miRNAs in histone modifications-related diseases.

## Materials and methods

### Sample collection

All the healthy individuals included were examined to be free from systematic disease history, smoking, alcoholic, and/or drug abuse history and had no tooth decay and/or acute or chronic oral infections. Twenty teeth of periodontitis patients were collected from the teeth extracted due to clinical diagnosed chronic periodontitis.

### Experimental animals

All animal experiments were conducted in accordance with the committee guidelines of the Fourth Military Medical University (FMMU), Xi’an, China and met the NIH guidelines for the care and use of laboratory animals. Sprague–Dawley (SD) rats and immunocompromised mice were obtained from the Laboratory Animal Research Centre of the Fourth Military Medical University. SD rats were used to construct the experimental periodontitis model. Immunocompromised 8-week-old mice were used as hosts for implantation.

### Isolation and culture of human PDLSCs

All experimental protocols were approved by the Committee of Ethics of Fourth Military Medical University and informed consent was obtained from each subject. The age of the participants ranged from 18 to 50 years. The healthy and inflammatory teeth were got from the school of stomatology of the FMMU. Human PDLSCs were isolated and cultured according to previously published procedures^33^.

### 3-2, 5-Diphenyltetrazolium bromide (MTT) dye reduction assays

The influence of the HDAC inhibitor (NaB) on the proliferative potential of human PDLSCs was investigated by the tetrazolium salt [MTT, 3-(4,5-dimethylthiazol-2-yl)-2,5-diphenyltetrazolium bromide] assay for 7 days. PDLSCs were seeded in 96-well plates at a density of 2 × 10^3^ cells/well and cultured in α-MEM (10% FBS) with 100 μM and 200 μM NaB at 37 °C in a humidified atmosphere containing 5% CO_2_. When the cells were adherent, 20 μl 5 mg/ml MTT (Sigma-Aldrich) solution was added to each well, and the plates were incubated for 4 h at 37 °C. Then, the medium was replaced with 200 μl DMSO (Sigma-Aldrich), and the absorbance was measured at 490 nm by a microplate reader (ELx800, BioTek Instruments Inc., USA).

### RNA interference

Small interfering RNA (siRNA) targeting HDAC9 and scram-bled siRNA were purchased from Santa (USA). The siRNA duplexes (final concentration 50 nM) were transfected into PDLSCs using Lipofectamine 2000 (Invitrogen, San Diego, CA, USA) according to the instructions. Cells were harvested 24 and 48 h later, respectively, and the knockdown efficiency was further determined via real-time PCR and Western blot analyses.

### Western blot analysis

Total protein lysates were obtained from harvested cells in protein RIPA lysis buffer (Beyotime, Shanghai, China) containing 10 mM phenylmethylsulphonyl fluoride as a protease inhibitor (PMSF; Beyotime) on ice for 30 min. The concentrations of total proteins were measured via Coomassie blue staining. The total proteins were separated on 10% SDS-PAGE gels and transferred to 0.45 m Immobilon-P Transfer Membranes (Millipore Corporation, Billerica, MA, USA). The membranes were blocked in 5% skim milk dissolved in Tris-buffered saline containing Tween and then immunoblotted with primary antibodies. Antibodies used in this experiment include: β-actin, GAPDH, RUNX2, ALP, OCN, SP7, PPAR-r (Abcam plc, Cambridge, UK), HDAC9, H3K9, H3K14, H3K18, H4K16 (R&D Systems, Minneapolis, MN, USA), and HRP-conjugated secondary antibodies to rabbit and mouse (Santa Cruz Inc., CA, USA).

### Alizarin red staining and ALP assay

Human PDLSCs in different groups were plated in 12-well plates at a density of 2 × 10^5^ cells/well and cultured in osteogenic medium. The mineralization potential of the cells was assessed via Alizarin red staining when cells were cultured with osteogenic medium for 28 days. The ALP enzymatic activities analysis was measured by ALP staining after osteogenic induction for 7 days according to the instruction of the ALP straining kit (Bi Yuntian, China).

### Real-time quantitative-PCR analysis

Reverse transcription (RT) and real-time quantitative PCR (qPCR) were performed using the PrimeScript RT reagent kit and real-time SYBR Premix Ex Taq^TM^ kit (TaKaRa Holdings Inc., Kyoto, Japan). The primers for each genes of interest were synthesized according to the sequences listed in Supplemental Table. All values were normalized to GAPDH (glyceraldehyde 3-phosphate dehydrogenase) or U6. And the relative quantification of gene expression was performed using the 2-∆∆CT method.

### Chromatin immunoprecipitation (ChIP)

The ChIP analysis was carried out using EZ-ChIP (Millipore, Billerica, MA, USA). Rabbit IgG was used as a negative control. Briefly, PDLSCs were cross-linked in 1% formaldehyde for 10 min and lysed in SDS lysis buffer and then sonicated to shear DNA. Lysates after diluted with ChIP dilution buffer were immunoprecipitated with rabbit IgG and HDAC9 antibody overnight at 4 °C. Antibody–chromatin complexes were precipitated with ChIP blocked protein G agarose for 1 h at 4 °C and then washed and eluted. After reverse crosslink of protein–DNA complexes, DNA was purified using spin columns and analyzed by real-time PCR.

### Paraffin-embedded immunohistochemistry (IHC-P)

All samples were fixed in 4% paraformaldehyde and then decalcified with 5% EDTA, followed by paraffin embedding. The paraffin sections were cut into 4 µm specimens, deparaffinized in series of xylene and rehydrated in grading alcohol solutions. Antigen retrieval is accomplished using autoclave heating for 2 min in citrate buffer (Heart Biological Technologies). The endogenous peroxidase activity is blocked using 3% H_2_O_2_. The specimens were incubated in 1.5% goat serum (Millipore) for 40 min at room temperature and then incubated with primary antibody anti-RUNX2 (Abcam) overnight at 4 °C, following horseradish peroxidase- conjugated secondary antibody incubation (Jackson Lab). The slides were incubated with 3,3-diaminobenzidine for 10 s. The nuclei were stained with hematoxylin for 20 s and were differentiated by 1% hydrochloric alcohol for 3 s. Following ddH_2_O incubation for 30 min, the specimens were dehydrated in serious of alcohol, xylene and preserved using natural balsam. Finally, the specimens were mounted for evaluation. Immunohistochemistry image analysis toolbox plugin downloaded from https://imagej.nih.gov/ij/plugins/ihc-toolbox/index.html is installed in ImageJ for automatic color detection of IHC-P images. H-DAB mode is used. For statistical quantification of the signal, the brown-signal-only images is transformed to RGB stack, automatically adjusted threshold and measured for percentage of area of signal vs. total area.

### Animal assay

#### Mouse subcutaneous implant model

PDLSCs treated with HDAC inhibitor (NaB 100 μM) respectively or with negative control were mixed with 40 mg HA-TCP ceramic particles (the National Engineering Research Center for Biomaterials of Sichuan University, China) and subcutaneously implanted into the dorsal surface of 8-week-old immunocompromised mice. At 8 weeks after implantation, the implants were harvested. For histological analysis, the implanted samples were fixed in 10% neutral buffered formalin (Sigma) overnight and embedded into paraffin. Sections were deparaffinized and stained with hematoxylin and eosin (H&E) following the procedures previously described^34^. The results were observed under a light microscope (BX-51, Olympus, Japan), and images were acquired using a CDD camera. The histological sections were analyzed using the National Institutes of Health ImageJ software.

#### SD rat experimental periodontitis model

The experimental periodontitis was inducted as previously described^35^. Twelve 8-week-old SD rats were randomly distributed into four groups (three rats per group): Saline; LPS (1 mg/ml); NaB (20 mg/ml). 10 μl drugs were injected in each group into the maxillary palatal gingiva between the first and second upper molars and were repeated every other day on 3 separate days. All rats were anesthetized and euthanized by exsanguination on day 7.

#### Micro-CT analysis

The whole head of SD rats were removed and the maxillary jaws were scanned and analyzed using a micro-CT system (Siemens Inveon Micro CT, Munich, Germany). After the mandibles were scanned, rebuilt images of bone surface were used to perform three-dimensional histomorphometric analysis with the same density. From rebuilt images, the alveolar bone height was measured at four different sites in two molars by recording the distance from the cemento-enamel junction to the alveolar bone crest. The distances were assessed as the mean distance of experiment groups were compared with the saline group with statistics.

### Statistical analysis

All experiments were repeated at least three times and data are presented as mean ± SD. Statistical significance was analyzed using SPSS 11.0 software (SPSS) and *p* < 0.05 was considered statistically significant.

## Electronic supplementary material

Supplementary figure legends

Supplementary figures
