# Supplementary material for: Mutual inhibition between HDAC9 and miR-17 regulates osteogenesis of human periodontal ligament stem cells in inflammatory conditions
Source: Cell Death Dis. 2018 Apr 24;9(5):480. doi: 10.1038/s41419-018-0480-6 (PMC5915523; doi:10.1038/s41419-018-0480-6)
Supplement: Supplementary file 1 — Supplementary figure legends [file 41419_2018_480_MOESM1_ESM.docx]

**Supplementary Figure 1**. Flow cytometry analysis of surface markers of PDLSCs. H-PDLSCs (A) and P-PDLSCs (B) was identified with MSC surface markers positive for CD90, CD105, CD146, negative for CD34, CD45.

**Supplementary Figure 2**. Effects of NaB on PDLSC proliferation analyzed by the MTT assay. 100μM/200μM NaB treated H-PDLSCs (A-C) or P-PDLSCs (D-F) exhibited no significant different OD value in the methyl thiazolyl tetrazolium (MTT) assay.

**Supplementary Figure 3**. Comparison of proliferation rate between the P-PDLSCs and H-PDLSCs by the MTT assay.

**Supplementary Figure 4**. Effects on PDLSC morphology with HDAC inhibitors NaB. (a) 100μM/200μM NaB exhibited no significant influence on the morphology of H-PDLSC compared with the control group. Scale bar represents 100μm. (b) 100μM/200μM NaB exhibited no significant influence on the morphology of P-PDLSC compared with the control group. Scale bar represents 100μm.

**Supplementary Figure 5**. Alizarin red staining of H-PDLSCs, P-PDLSCs and NaB treated P-PDLSCs. Scale bars represent 100μm.

**Supplementary Figure 6**. Comparison of protein expression level of HDAC9 in H-PDLSCs and P-PDLSCs.

**Supplementary Figure 7**. Selection of the appropriate concentration of HDI.
