# Supplementary material for: Mutual inhibition between HDAC9 and miR-17 regulates osteogenesis of human periodontal ligament stem cells in inflammatory conditions
Source: Cell Death Dis. 2018 Apr 24;9(5):480. doi: 10.1038/s41419-018-0480-6 (PMC5915523; doi:10.1038/s41419-018-0480-6)
Supplement: Supplementary file 2 — Supplementary figures [file 41419_2018_480_MOESM2_ESM.pdf]

Figure S1

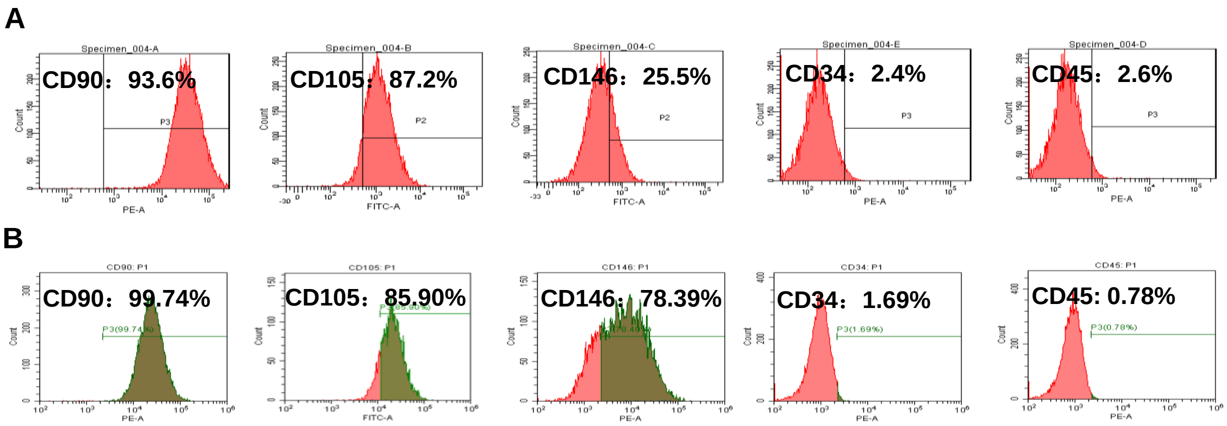
A
CD90：93.6%
CD105：87.2%
CD146：25.5%
CD34：2.4%
CD45：2.6%
B
CD90：99.74%
CD105：85.90%
CD146：78.39%
CD34：1.69%
CD45: 0.78%

Figure S2

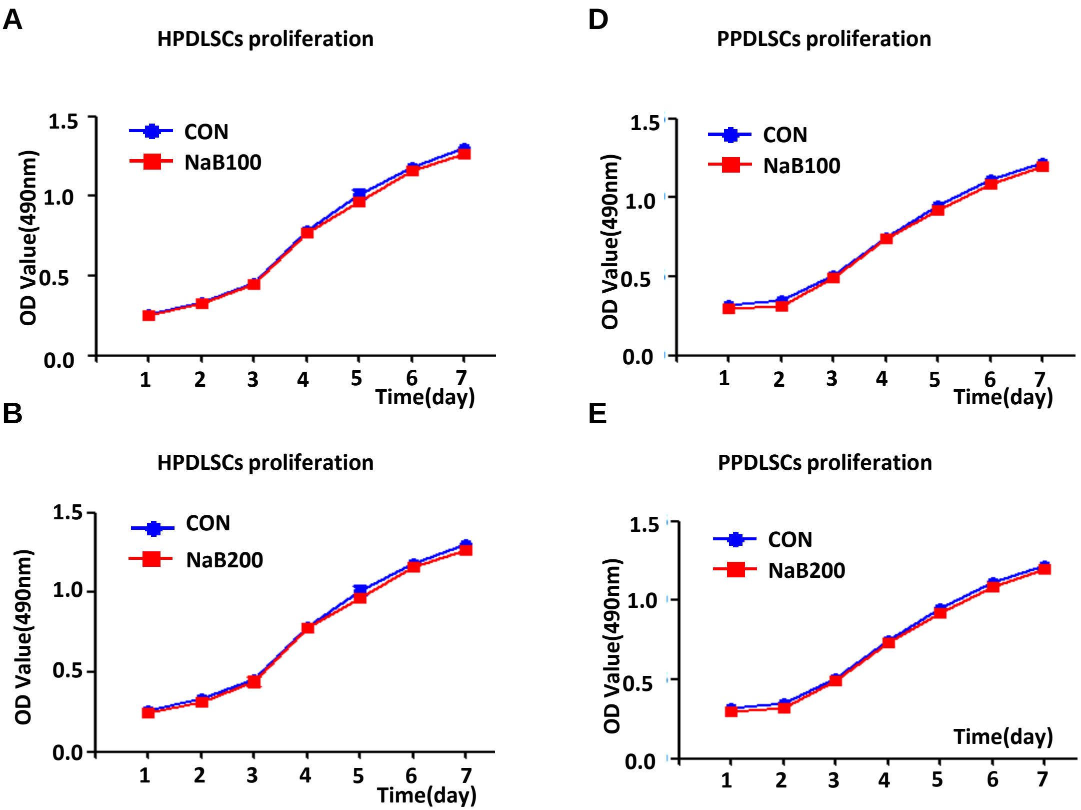
A
HPDLSCs proliferation
OD Value(490nm)
1.5
1.0
0.5
0.0
CON
NaB100
1
2
3
4
5
6
7
Time(day)
B
HPDLSCs proliferation
OD Value(490nm)
CON
NaB200
Time(day)
D
PPDLSCs proliferation
OD Value(490nm)
CON
NaB100
Time(day)
E
PPDLSCs proliferation
OD Value(490nm)
CON
NaB200
Time(day)

Figure S3

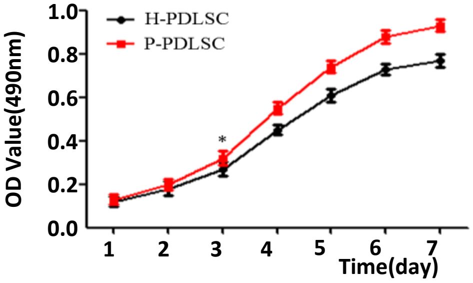
H-PDLSC
P-PDLSC
OD Value(490nm)
1.0
0.8
0.6
0.4
0.2
0.0
*
1
2
3
4
5
6
7
Time(day)

Figure S4

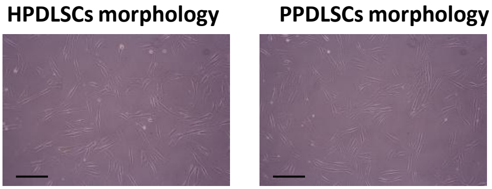

Figure S5

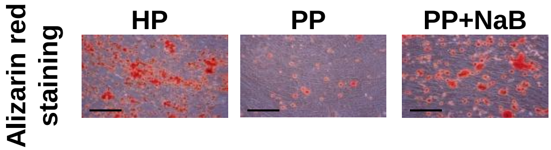

Figure S6

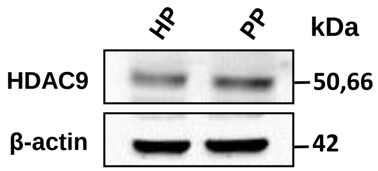

Figure S7

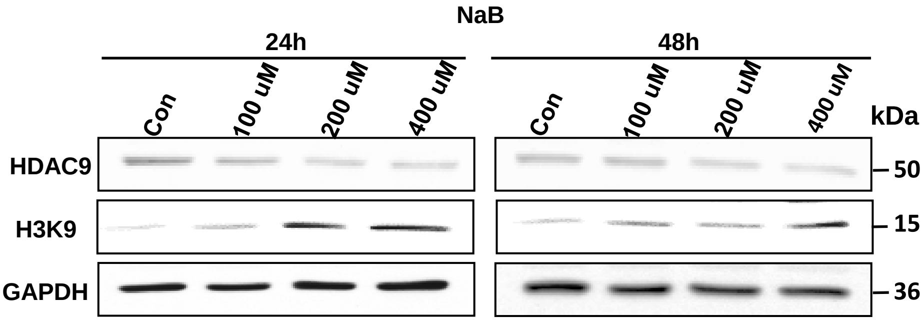
NaB
24h
48h
Con
100 uM
200 uM
400 uM
Con
100 uM
200 uM
400 uM
kDa
HDAC9
H3K9
GAPDH
50
15
36
